# One-step process for production of *N*-methylated amino acids from sugars and methylamine using recombinant *Corynebacterium glutamicum* as biocatalyst

**DOI:** 10.1038/s41598-018-31309-5

**Published:** 2018-08-27

**Authors:** Melanie Mindt, Joe Max Risse, Hendrik Gruß, Norbert Sewald, Bernhard J. Eikmanns, Volker F. Wendisch

**Affiliations:** 1 Genetics of Prokaryotes, Faculty of Biology & CeBiTec, Bielefeld University, Universitätsstr. 25, 33615 Bielefeld, Germany; 2 Fermentation Technology, Technical Faculty & CeBiTec, Bielefeld University, Universitätsstr. 25, 33615 Bielefeld, Germany; 3 Organic Chemistry III, Faculty of Chemistry & CeBiTec, Bielefeld University, Universitätsstr. 25, 33615 Bielefeld, Germany; 4 Institute of Microbiology and Biotechnology, University of Ulm, Albert-Einstein-Allee 11, 89081 Ulm, Germany

## Abstract

*N*-methylated amino acids are found in Nature in various biological compounds. *N*-methylation of amino acids has been shown to improve pharmacokinetic properties of peptide drugs due to conformational changes, improved proteolytic stability and/or higher lipophilicity. Due to these characteristics *N*-methylated amino acids received increasing interest by the pharmaceutical industry. Syntheses of *N*-methylated amino acids by chemical and biocatalytic approaches are known, but often show incomplete stereoselectivity, low yields or expensive co-factor regeneration. So far a one-step fermentative process from sugars has not yet been described. Here, a one-step conversion of sugars and methylamine to the *N*-methylated amino acid *N*-methyl-l-alanine was developed. A whole-cell biocatalyst was derived from a pyruvate overproducing *C. glutamicum* strain by heterologous expression of the *N*-methyl-l-amino acid dehydrogenase gene from *Pseudomonas putida*. As proof-of-concept, *N*-methyl-l-alanine titers of 31.7 g L^−1^ with a yield of 0.71 g per g glucose were achieved in fed-batch cultivation. The *C. glutamicum* strain producing this imine reductase enzyme was engineered further to extend this green chemistry route to production of *N*-methyl-l-alanine from alternative feed stocks such as starch or the lignocellulosic sugars xylose and arabinose.

## Introduction

*N*-alkylation of amino acids occur in bacteria and eukaryotes. In green tea leaves, the *N*^5^-ethylated L-glutamine derivative theanine was shown to be responsible for their umami taste^1,2^. *N*-methylated amino acids are also found in depsipeptides that are used as drugs e.g. vancomycin, actinomycin D and cyclosporine. *N*-methylamino acid containing peptides often show higher stability against proteolytic degradation and/or increased membrane permeability as compared to non-methylated peptides^3–5^. Accordingly, the substitution of an *N*-terminal glycine residue for sarcosine in an angiotensin II analog enhanced *in vivo* activity as a potential result of longer half-life against proteolytic degradation^6^. Similar to l-proline, *N*-methylated amino acids are known to stabilize discrete conformations of peptides as shown for the exchange of l-pipecolic acid by *N*-methyl-l-alanine in the ATPase inhibitor efrapeptin C^7^.

**Figure 1:**
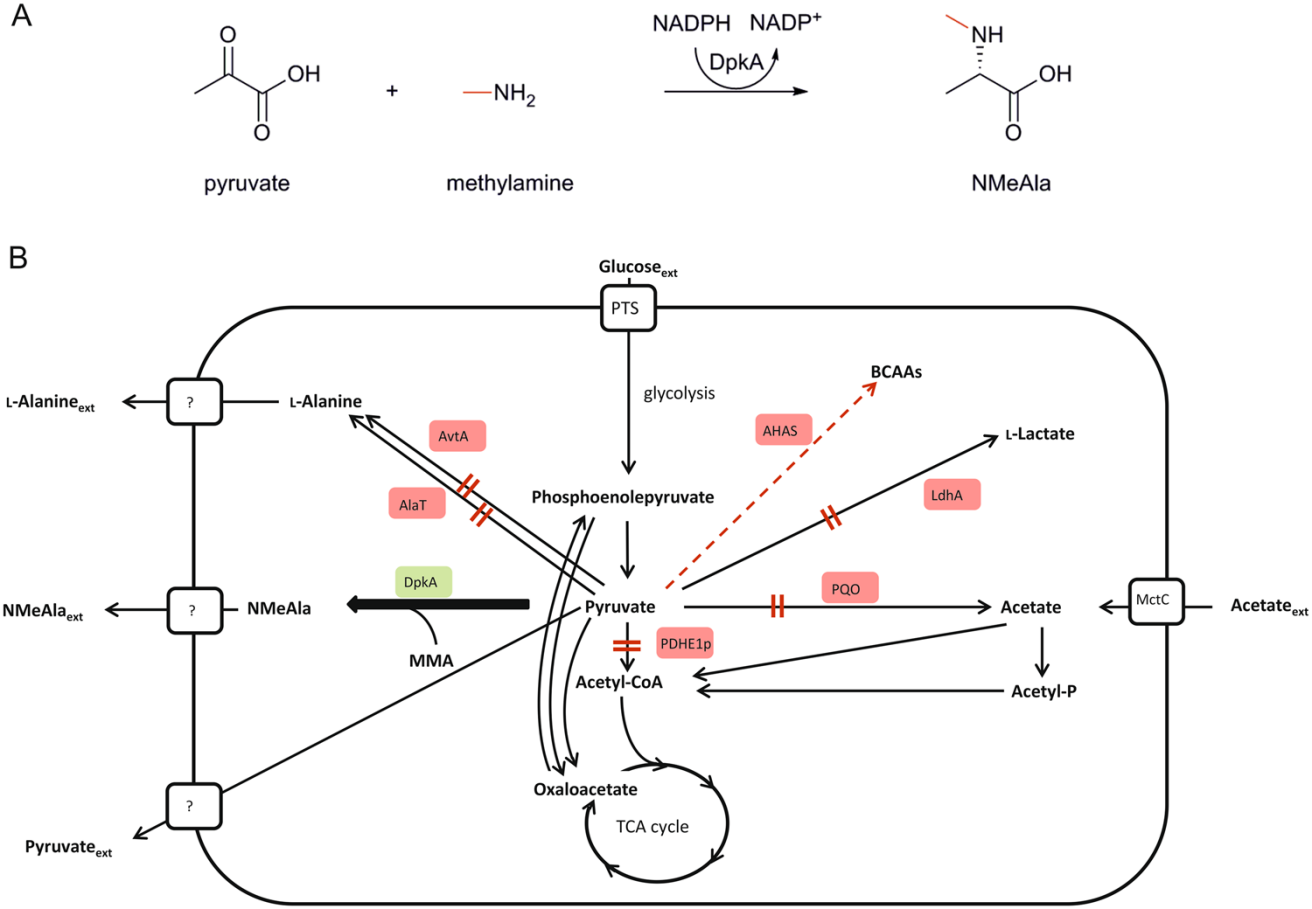
Schematic overview of the reaction catalyzed by DpkA (**A**) and its integration into the central carbon metabolism in *C. glutamicum* NMeAla1 (**B**). The gene deletions for improved pyruvate production are shown by black arrows with red double bars: deletion of *aceE* (encoding PDHE1p, the E1p subunit of the PDHC) and *pqo* (encoding pyruvate-quinone oxidoreductase, PQO) and both genes coding both major enzymes for l-alanine supply by pyruvate aminotransferases (*alaT* and *avtA*, encoding the alanine aminotransferase AlaT and the valine-pyruvate aminotransferase AvtA, respectively) were deleted. In addition, the acetohydroxyacid synthase (AHAS) activity was downregulated by deletion of the C-terminal part of *ilvN* (small subunit of AHAS) shown by red dashed arrow. Enzymes highlighted by red background indicate missing or down regulated enzymes. The thick arrow displays the NMeAla formation by heterologous expressed *dpkA* from *P. putida* KT2440 coding for the *N*-methylated amino acid dehydrogenase DpkA (green shadowed Enzyme).

In certain bacteria, utilization of mono-methylamine (MMA) may lead to *N*-methylated amino acids. Some bacteria that can grow with reduced carbon substrates without carbon-carbon bonds such as methane or methanol can utilize MMA as sole source of carbon. The *N*-methylated amino acid *N*-methylglutamate occurs as an intermediate of the so-called monomethylamine catabolic pathway in representatives of these methylotrophic bacteria, e.g. *Methylocella silvestris*, *Methyloversatilis universalis* or *Methylobacterium extorquens*^8–11^. In cell free extracts of *Pseudomonas* MS *N*-methylalanine (NMeAla) was observed when MMA was added to the growth medium^12^. An enzyme which catalyzes the reductive methylamination of pyruvate to NMeAla in the presence of MMA was isolated and named *N*-methylalanine dehydrogenase (Fig. 1)^13^. Based on its native function of reducing piperideine-2-carboxylate in addition to the asymmetric synthesis of chiral amines this enzyme belongs to the class of imine reductases (IREDs)^14,15^. The corresponding gene *dpkA* from *Pseudomonas putida* ATCC12633 was identified and characterization of the encoded enzyme revealed a somewhat relaxed substrate spectrum. Since α-keto acids such as phenylpyruvate, ketohexanoate and ketoisobutyrate were accepted aside from pyruvate and the enzyme also converts other alkylamines such as *N*-ethylamine, it was named *N*-methyl-l-amino acid dehydrogenase or NMAADH^16–18^. Reductive alkylamination of α-keto acids by DpkA using MMA appears similar to reductive amination by amino acid dehydrogenases using ammonium. Yet, the structure of DpkA shows similarities to a new subclass of Nicotinamide adenine dinucleotide phosphate (NADPH)-dependent oxidoreductases with the rare SESAS (seven-stranded predominantly antiparallel β-sheet) fold for NADPH-binding^19^. The physiological role of NMAADH activity in pseudomonads, however, remains elusive.

Although the formation of *N*-alkylated amino acids such as NMeAla has been shown during the characterization of NMAADH^16–18^, efficient production via biocatalysis or by fermentation has not yet been described. Biocatalytic approaches may offer advantages over chemical methods such as *N*-alkylation of amino acids or the asymmetric Strecker synthesis since the chemical methods may use hazardous chemicals, give only incomplete stereoselectivity and low yields, while side reactions like dimethylation of the amino group may occur^20^.

Fermentative production of amino acids, mainly l-glutamate and l-lysine, occurs at the million-ton-scale^21^. For more than fifty years, *C. glutamicum* has been used for the safe production of food and feed amino acids^22,23^. Besides the flavor enhancing l-glutamate^24^ and the feed additive l-lysine^25^, further amino acids and related compounds can efficiently be produced by glucose- and ammonium-based fermentation using recombinant *C. glutamicum* strains^26,27^. Metabolic engineering of *C. glutamicum* has not been restricted to amino acids but also production of the α-keto acids pyruvate, ketoisovalerate and ketoisocaproate^28–31^ were established. Taking the broad substrate range of the NMAADH from *P. putida* into account, it is important to engineer a host such as *C. glutamicum* to overproduce only one α-keto acid, in this study pyruvate.

Here we describe the one-step production of the *N*-methylated amino acid NMeAla from glucose and methylamine by a newly constructed *C. glutamicum* whole cell biocatalyst. The NMAADH gene *dpkA* from *P. putida* was expressed in a pyruvate overproducing *C. glutamicum* strain^31^. This pyruvate producing strain, named ELB-P, is able to secrete up to 17.6 g L^−1^ pyruvate with low by-product formation in shake flasks^31^. To achieve high titers of pyruvate, the genome of this strain carries deletions of the genes encoding pyruvate-converting enzymes. Starting with a pyruvate dehydrogenase gene (*aceE* encoding the E1p subunit) deficient strain^32^, which accumulates high titers of pyruvate^33^, additional deletion of the pyruvate-quinone oxidoreductase gene (*pqo*)^34^ and deletion of the C-terminal regulatory domain of the acetohydroxyacid synthase gene (*ilvN*)^35,36^ further increased pyruvate availability. To prevent the reduction of pyruvate to lactic acid, the *ldhA* (NAD-dependent l-lactic acid dehydrogenase)^35^ was deleted. Additionally, formation of the by-product l-alanine was reduced by deletion of the alanine aminotransferase gene (*alaT*) and valine-pyruvate aminotransferase gene (*avtA*)^37^ (Fig. 1). *C. glutamicum* ELB-P requires acetate for biomass formation as consequence of the *aceE* deletion and uses glucose for production of pyruvate^31,38^. A derivative of *C. glutamicum* ELB-P expressing *dpkA* from *P. putida* was constructed here and demonstrated to be suitable for the one-step production of NMeAla from MMA and glucose or alternative feedstocks.

## Results

### *Corynebacterium glutamicum* as suitable host for NMeAla production

To determine if *C. glutamicum* is a suitable host for the production of the *N*-methylated amino acid NMeAla, the growth behavior of the wild type strain was analyzed under different conditions. To test whether *C. glutamicum* is able to utilize MMA or NMeAla as sole carbon or nitrogen source it was grown in minimal medium with either 50 mm MMA, NMeAla or glucose as sole carbon source or with either 50 mm MMA, 50 mm NMeAla or 30 mm ammonium sulfate and 17 mm urea as nitrogen source. This growth experiment revealed that *C. glutamicum* could neither use MMA nor NMeAla as sole carbon or nitrogen source (data not shown).

**Figure 2:**
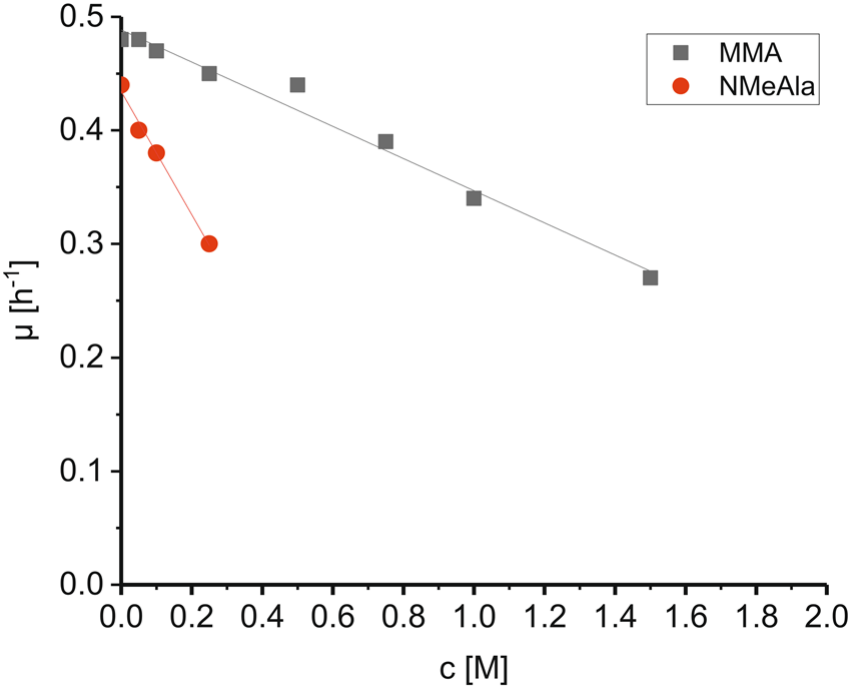
Growth rates of *C. glutamicum* wild type in the presence of varying concentrations of MMA or NMeAla. *C. glutamicum* wild type was grown in presence of increasing MMA (0.05 m to 1.5 m) or NMeAla (0.05 m to 0.25 m) concentrations and specific growth rates were determined. Half maximal growth rates were obtained by extrapolation.

Possible effects due to substrate or product toxicity were detected in growth experiments with *C. glutamicum* wild type in minimal medium with glucose and increasing concentrations of MMA (0.05 m to 1.5 m) or NMeAla (0.05 m to 0.25 m). The growth rate was diminished at higher concentrations to about half-maximal rates at 1.8 m MMA and 0.4 m NMeAla, respectively (Fig. 2). In order to determine if MMA affects global gene expression in *C. glutamicum*, the transcriptomes were compared during growth in glucose minimal medium containing either 250 mm MMA or 125 mm ammonium sulfate. The finding that very few genes changed expression and none had a function in nitrogen metabolism (Supplementary Table) indicated that MMA does not elicit a specific gene expression response.

### Metabolic engineering of *C. glutamicum* for fermentative production of NMeAla

The relatively small impacts of the substrate MMA and the product NMeAla on growth make *C. glutamicum* a suitable host organism for the fermentative production of NMeAla if a) sufficient pyruvate is available and b) NMeAla is transported out of the *C. glutamicum* cell. Therefore, the pyruvate overproducing *C. glutamicum* strain ELB-P^31^ was chosen as platform strain for engineering fermentative production of NMeAla. Since NMAADH activity had not been reported for *C. glutamicum*, the NMAADH gene *dpkA* from *P. putida* was cloned into the expression vector pVWEx1 and used to transform *C. glutamicum* ELB-P (Fig. 1). The resulting strain ELB-P(pVWEx1-*dkpA*) was designated as NMeAla1. Crude extracts of cells carrying either the empty vector or the *dpkA* expression vector were assayed for reductive *N*-methylamination of pyruvate. As presumed, no activity was detected for *C. glutamicum* carrying the empty vector whereas a specific activity of 24 ± 1 mU (mg protein^−1^) for reductive *N*-methylamination of pyruvate was detected in the *dpkA* expressing strain. This result indicates functional expression of *dpkA* from *P. putida* in *C. glutamicum*.

**Figure 3:**
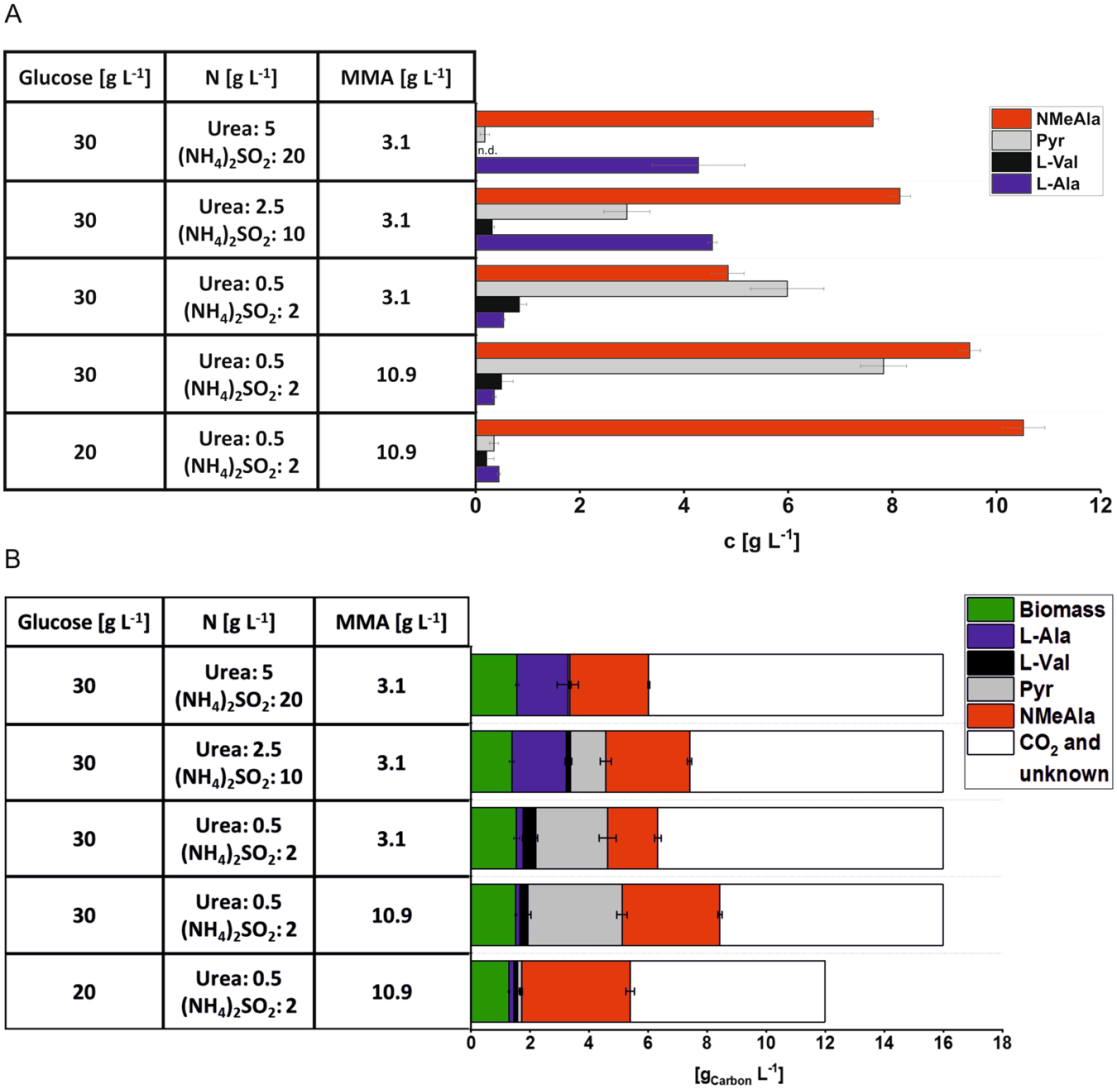
NMeAla, l-alanine, l-valine and pyruvate production data (**A**) and carbon balance (**B**) of *C. glutamicum* strain NMeAla1 under different conditions. Cells were cultivated in minimal medium CGXII containing 30 g L^−1^ or 20 g L^−1^ glucose and 16.6 g L^−1^ potassium acetate, 2 mm l-Ala and 1 mm IPTG for induction of gene expression. The nitrogen amount of the minimal medium was reduced to 50**%** or 10**%** respectively, the glucose and MMA amount were optimized to finally 20 g L^−1^ glucose and 10.9 g L^−1^ MMA. The culture supernatants were harvested after incubation for 72 h and analyzed by HPLC. (**A**) Concentrations are given as means with standard deviation of three replicates. n.d.: not detected. (**B**) To assess the fate of carbon from glucose and acetate as substrates their concentrations in gram carbon per liter is plotted. The gram carbon per liter concentrations of biomass formed (green) and of the formed products l-alanine (blue), l-valine (black), pyruvate (grey), and NMeAla (red) are plotted. For NMeAla, the carbon derived from MMA was not considered. The gram carbon per liter concentrations of CO_2_ and unknown byproducts are depicted in open columns. Amines < 0.1 g L^−1^ and carbohydrates < 0.5 g L^−1^ were not considered.

In order to test *C. glutamicum* strain NMeAla1 for NMeAla production, the strain was cultivated in minimal medium supplemented with 16.6 g L^−1^ potassium acetate, 2 mm l-Ala, 30 g L^−1^ glucose and 3.1 g L^−1^ MMA. HPLC analysis of supernatants after cultivation for 72 h revealed that *C. glutamicum* NMeAla1 produced 7.6 ± 0.1 g L^−1^ NMeAla (Fig. 3). Only 0.2 ± 0.1 g L^−1^ pyruvate were produced, but the by-product l-alanine accumulated to considerable concentrations (4.3 ± 0.9 g L^−1^; Fig. 3).

### Improvement of precursor conversion and reduction of by-product formation

The formation of l-alanine as by-product may be due to the high concentrations of ammonium sulfate and urea present as nitrogen sources in CGXII minimal medium. CGXII minimal medium was optimized for production of l-lysine which contains two ammonium groups and reducing the nitrogen content in CGXII medium has previously been shown to improve production of l-proline and γ-aminobutyric acid that only contain a single ammonium group^39,40^. For production of NMeAla, MMA is used for reductive *N*-methylamination of pyruvate while ammonium sulfate and urea are required solely to support biomass formation. Therefore, the nitrogen amount of the minimal medium was reduced by half (2.5 instead of 5 g L^−1^ urea and 10 instead of 20 g L^−1^ ammonium sulfate) and to 10% (0.5 instead of 5 g L^−1^ urea and 2 instead of 20 g L^−1^ ammonium sulfate). Under the latter condition formation of the by-product l-alanine was diminished, however, increased pyruvate concentrations and decreased NMeAla concentrations in the supernatants indicated incomplete reductive *N*-methylamination of pyruvate to NMeAla (Fig. 3A). Subsequently, the MMA concentration was increased to 10.9 g L^−1^ and in addition the glucose concentration was reduced to 20 g L^−1^. As a result, only low concentrations of pyruvate, l-alanine and l-valine accumulated as by-products while a titer of 10.5 ± 0.4 g L^−1^ of NMeAla was obtained within 72 h (Fig. 3). To obtain an idea of the fate of carbon from glucose and acetate as substrates the concentrations of carbon present in the biomass and products formed were plotted (Fig. 3B). While *E. coli* shows overflow metabolism at high glucose concentrations, *C. glutamicum* does not^22,23^. Specifically, the strain used here did neither secrete acetate nor lactate due to gene deletions introduced by metabolic engineering (Δ*aceE*, Δ*pqo*, Δ*ldhA)*. As expected for aerobic processes, about 50% of carbon from the growth substrates will end up in CO_2_. For example under the condition with 20 g L^−1^ glucose and 16.6 g L^−1^ potassium acetate (together 12 g carbon L^−1^), 11% carbon was found in biomass, 2% in L-alanine, 1% in L-valine, 1% in pyruvate and 31% NMeAla, while CO_2_ formation likely explains the fate of 55% of the carbon.

Thus, after balancing concentrations of the nitrogen and carbon sources for growth (ammonium sulfate, urea and acetate) with the substrates for production (glucose and MMA), NMeAla was produced by fermentation using *C. glutamicum* strain ELB-P(pVWEx1-*dkpA*) with a volumetric productivity of 0.15 g L^−1^ h^−1^ and a yield of 0.53 g g^−1^ glucose.

### Fed-Batch bioreactor process of NMeAla production

**Figure 4:**
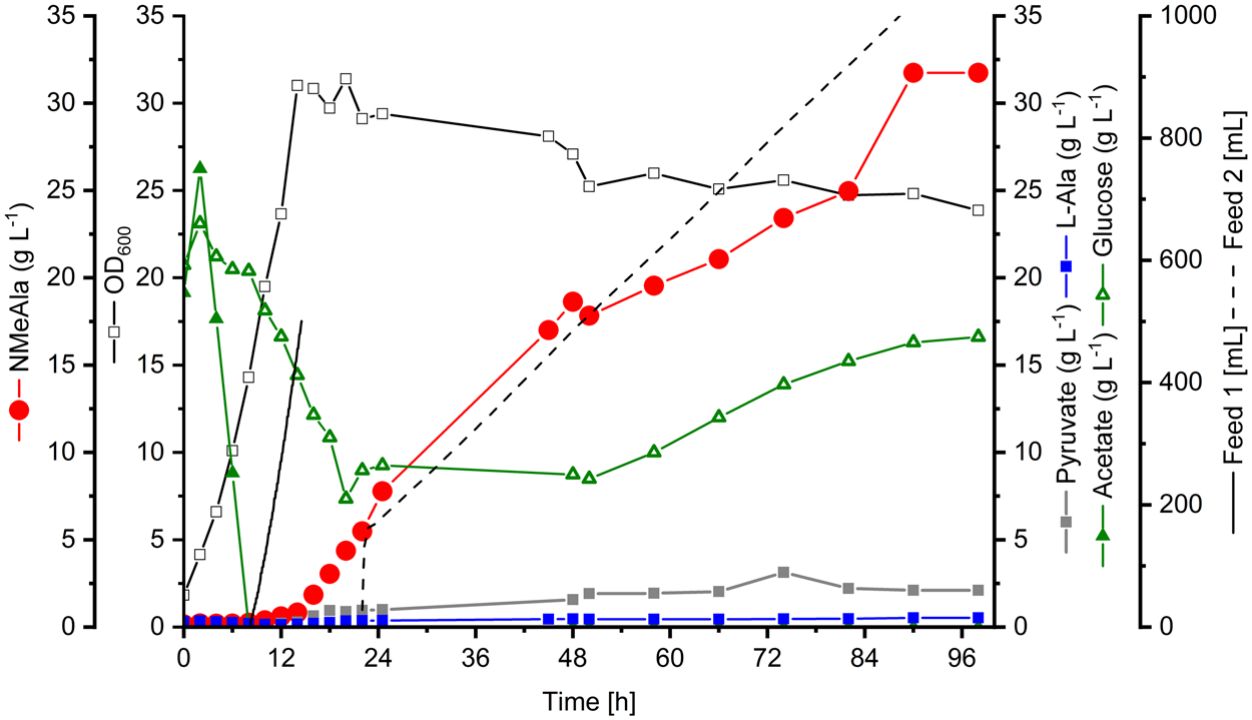
Fed-batch cultivation with *C. glutamicum* NMeAla1 in minimal medium supplemented with potassium acetate and glucose as carbon and energy sources. A fermenter with an initial start volume of 4 L was used. First feed phase (potassium acetate) was coupled to the rDOS value. After 22 h the second feed phase was started by the initial addition of 162 mL of a glucose/MMA solution followed by a linear feed of 12.3 mL h^−1^. The biomass formation (black open squares), concentrations of NMeAla (red circles), l-alanine (blue squares), pyruvate (grey squares), acetate (green filled triangles) and glucose (green open triangles) were depicted. The volume of both feeds is shown as black lines. All depicted concentrations and the biomass formation was related to the initial volume.

To evaluate an enhancement of NMeAla production by feeding glucose and MMA a fed-batch cultivation in 4 L scale (initial volume) was performed. For higher cell density and higher production titers the fed-batch cultivation was performed with two independent feed phases (Fig. 4). The first feeding solution contained acetate and was coupled to the relative dissolved oxygen saturation (rDOS) signal with the intent to increase the biocatalyst concentration and to improve growth-associated production of NMeAla. The second feeding phase started after 22 h with an initial supply of 162 mL followed by a linear feed (12.3 mL h^−1^) of glucose and MMA (ratio 1:3) to boost growth-decoupled production of NMeAla. At the end of the fed-batch bioreactor process (98 h) 86.7 g acetate and 178.8 g glucose were consumed and the residual glucose concentration was 16.3 g L^−1^. A yield of 0.48 g NMeAla per g of acetate and glucose was achieved. Considering that growth of *C. glutamicum* NMeAla1 depends on acetate whereas production does not, a product yield on glucose was calculated to be 0.71 g g^−1^ glucose at a final titer of 31.7 g L^−1^ NMeAla and a volumetric productivity of 0.35 g L^−1^ h^−1^. The side-product l-alanine and the precursor pyruvate only accumulated to low concentrations (0.5 g L^−1^ and 2.1 g L^−1^, respectively). Thus, fermentative production of NMeAla in a fed-batch process resulted in enhanced final titer, volumetric productivity and yield in comparison to shake flask experiments.

### Establishing production of NMeAla from alternative feedstocks

**Figure 5:**
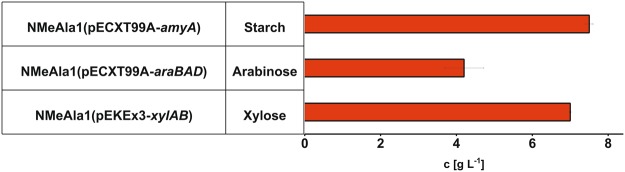
Production of NMeAla from alternative carbon sources. The CGXII minimal medium with 16.6 g L^−1^ potassium acetate contained 30 g L^−1^ starch for cultivation production experiments using *C. glutamicum* strain NMeAla1(pECXT99A*-amyA*), 30 g L^−1^ arabinose using *C*. *glutamicum* strain NMeAla1(pECXT99A*-araBAD*) and 30 g L^−1^ xylose using *C*. *glutamicum* strain NMeAla1(pEKEx3-*xylAB*). Concentrations were determined after 72 h and are given as means with standard deviations of three replicates.

Sustainable production from sugars that have competing uses in human and animal nutrition have to be succeeded by production processes based on second generation feedstocks such as lignocellulosic hydrolysates. Fermentative production of amino acids is typically based on glucose present in molasses or obtained from starch by hydrolysis. Direct utilization of starch as well as of the pentose sugars xylose and arabinose that can be obtained by hydrolysis of lignocellulosics required metabolic engineering of *C. glutamicum*^41^. Based on these strategies the *C. glutamicum* strains NMeAla1(pEXCT99A-*amyA*), NMeAla1(pEKEx3-*xylA*_*Xc*_*-xylB*_*Cg*_), and NMeAla1(pEXCT99A-*araBAD*) were constructed and tested for production of NMeAla from starch, arabinose and xylose, respectively. Upon expression of the α-amylase gene *amyA* from *Streptomyces griseus, C. glutamicum* can utilize starch^42^ and *C. glutamicum* strain NMeAla1(pEXCT99A-*amyA*) produced 7.5 ± 0.1 g L^−1^ NMeAla in minimal medium containing 30 g L^−1^ starch and 16.6 g L^−1^ potassium acetate (Fig. 5). Heterologous expression of the arabinose utilization operon *araBAD* from *E. coli* enables *C. glutamicum* to utilize arabinose as carbon and energy source^43,44^. *C. glutamicum* strain NMeAla1(pEXCT99A-*araBAD*) produced 4.2 ± 0.5 g L^−1^ NMeAla in minimal medium containing 30 g L^−1^ arabinose and 16.6 g L^−1^ potassium acetate (Fig. 5). Efficient utilization of the lignocellulose pentose sugar xylose was enabled by expression of the xylose isomerase gene *xylA* from *Xanthomonas campestris* combined with overexpression of the endogenous xylulokinase gene *xylB*^45^. In CGXII minimal medium containing 30 g L^−1^ xylose and 16.6 g L^−1^ potassium acetate, *C. glutamicum* strain NMeAla1(pEKEx3-*xylAB*) produced 7.0 ± 0.1 g L^−1^ of NMeAla (Fig. 5). Taken together, efficient production of NMeAla from three alternative feedstocks was shown.

## Discussion

Fermentative access to the *N*-methylated amino acid NMeAla was achieved by introduction of the NMAADH gene *dpkA* from *P. putida* into the pyruvate overproducing *C. glutamicum* strain ELB-P. *N*-methyl-l-alanine titers of 31.7 g L^−1^ with a yield of 0.71 g per g glucose were achieved in fed-batch cultivation. The described *C. glutamicum* strains allow, to the best of our knowledge, the first fermentative production of NMeAla reported to date. *Di*-*N*-methyl-l-alanine, a frequent by-product in chemical methylation of l-alanine, was not observed. However, pyruvate and l-alanine accumulated as minor by-products.

l-Alanine was also a by-product of pyruvate^31^ and l-serine production^46^ although the genes alanine aminotransferase (*alaT*) and valine-pyruvate aminotransferase (*avtA*) have been deleted. Thus, at least one further l-alanine forming transaminase must be active in *C. glutamicum*.

Abolishing export of pyruvate and l-alanine was not possible since the export systems have not been identified. Deletion of export genes has been shown to be valuable to improve production of γ-aminobutyric acid (deletion of *cgmA* to abolish putrescine export)^47^, 5-aminovalerate and ectoine (deletion of lysine export gene *lysE*)^48–50^. As is the case for l-alanine, the export system for NMeAla is unknown. Given their similar structure it is conceivable that both l-alanine and NMeAla are exported by the same unknown export system. Alternatively, NMeAla may be substrate of the export system of *C. glutamicum* for branched-chain amino acids and l-methionine BrnEF^51–53^. The transcriptional regulator Lrp is activating transcription of *brnFE* at elevated intracellular concentrations of branched-chain amino acids and l-methionine^54^. Since *N*-methylation increases lipophilicity^55,56^, diffusion of the more lipophilic NMeAla across the cytoplasmic membrane of *C. glutamicum* is more relevant as compared to l-alanine. However, diffusion of amino acids across the cytoplasmic membranes of bacteria cannot explain transport processes against concentration gradients which require active transport systems. This not only holds true for charged amino acids such as l-lysine^57^, but also for uncharged amino acids such as l-isoleucine^51,52^. Future work will have to unravel the export systems of NMeAla, l-alanine and pyruvate in *C. glutamicum*.

In contrast, uptake of MMA into the *C. glutamicum* cell has been studied to some detail. The uncharged ammonia (NH_3_) is able to diffuse across the membrane, but its protonated form ammonium (NH_4_^+^) is actively imported by the transport proteins AmtA and AmtB^58,59^. Notably, as a probe for ammonium uptake, ^14^C-labelled MMA was used to determine uptake rates. Ammonium uptake in enteric bacteria such as *E. coli* operates by a comparable mechanism as shown for *E. coli* protein AmtB^60^.

The one-step process of NMeAla production will benefit from more efficient reductive *N*-methylamination of pyruvate by increasing the amount and/or the activity of NMAADH. Here, the NMAADH gene *dpkA* was cloned into the medium copy vector pVWEx1 and transcription initiated from the IPTG inducible promoter Ptrc and translation initiated from a standard ribosome binding site. Thus, as shown for expression of other genes in recombinant *C. glutamicum, dpkA* expression may benefit from the choice of the expression vector, the promoter and the ribosome binding site^61–64^.

Engineering of DpkA for more efficient reductive *N*-methylamination of pyruvate to yield NMeAla will also increase fermentative NMeAla production. The NMAADH DpkA used here for reductive *N*-methylamination of pyruvate has been shown to be part of the d-lysine degradation pathway in pseudomonads where it acts as imine reductase (IRED) reducing its native substrate piperideine-2-carboxylate^18^. IREDs that catalyze the asymmetric reduction of prochiral imines to chiral amines by using NAD(P)H as a hydride source are gaining increasing interest in bioorganic chemistry^65–68^. The substrate range is not restricted to cyclic imines and as shown for DpkA^16–18^, the (*S*)-selective IRED from *Streptomyces sp*. GF3546^69^ and the (*R*)-selective IRED from *Streptosporangium roseum*^70^ also catalyze asymmetric reductive amination from suitable ketone and amine precursors. The latter reaction is expected to proceed via an imine either in solution or in the active site of the enzyme. Structure-function analysis of DkpA and other IREDs to improve asymmetric reductive amination from suitable ketone and amine precursors has not yet been described, but would be valuable to increase reductive *N*-methylamination of pyruvate to NMeAla by DkpA or derived variants. This approach has successfully been applied to the P450 oxidoreductase BM3 from *B. megaterium*^71^. By mutagenesis the enzyme was engineered to oxidize not only fatty acids^72^, but also *N*-alkanes^73,74^, the more sterically demanding β-ionone^75^, indole^76,77^ and others.

Here, we have developed a fermentative route to the *N*-methylated amino acid NMeAla. The biocatalytic route was based on *N*-methyl-l-amino acid dehydrogenase (NMAADH), which was integrated into the central metabolism of a pyruvate overproducing *C. glutamicum* strain. A final NMeAla titer of 31.7 g L^−1^ was achieved in fed-batch fermentation after balancing the ratio of the major substrates glucose and MMA. Additionally, NMeAla production from the alternative carbon sources xylose, arabinose and starch was enabled, thus, providing the basis for sustainable NMeAla production from second generation feedstocks.

## Experimental

### Bacterial strains and growth conditions

The strains and plasmids used in this study are listed in Table 1. *E. coli* DH5α^78^ was used for vector construction. *C. glutamicum* pre-cultures were grown in Lysogeny Broth (LB) medium containing 7 g L^−1^ sodium acetate in 500 mL baffled flask at 30 °C inoculated from a fresh LB agar plate. When necessary, the medium was supplemented with kanamycin (25 µg mL^−1^), spectromycin (100 µg mL^−1^) and/or tetracyclin (5 µg mL^−1^). The gene expression from the vectors pVWEx1, pEKEx3 and pECXT99A was induced by adding Isopropyl-β-D-1-thiogalactopyranoside (IPTG) (1 mm). For growth experiments or fermentative production of *C. glutamicum* cells were incubated in LB medium containing 7 g L^−1^ sodium acetate overnight on a rotary shaker, harvested (4000 × g, 7 min) and washed with TN buffer pH 6.3 (50 mm TrisHCl, 50 mm NaCl). The cells were inoculated to an optical density at 600 nm (OD_600_) of 1 in 50 mL CGXII minimal medium^22^ supplemented with 40 g L^−1^ glucose (wild type) or with blends of 20 or 30 g L^−1^ glucose, 16.6 g L^−1^ potassium acetate and 2 mm L-alanine (ELB-P). Growth in 500 mL baffled flasks was followed by measuring the OD_600_ using V-1200 Spectrophotometer (VWR, Radnor, PA, USA). The Biolector microfermentation system (m2p-labs, Aachen, Germany) was used for determination of the growth behavior in the presence of MMA or NMeAla and the carbon and nitrogen source growth tests. The shaking frequency was adjusted to 1200 rpm and 48-well flower plate wells with cultivation volumes of 1 mL were used and growth was followed by backscattered light at 620 nm and a signal gain factor of 20.

**Table 1:** Bacterial trains and vectors used in this study.

Strains and vectors	Description	Source
**Strains**		
WT	*C. glutamicum* wild type, ATCC13032	American Type Culture Collection
ELB-P	WT carrying deletions *ΔaceE Δpqo ΔldhA ΔC-T ilvN ΔalaT ΔavtA*	^31^
NMeAla1	WT carrying deletions *ΔaceE Δpqo ΔldhA ΔC-T ilvN ΔalaT ΔavtA* and vector pVWEx1-*dpkA*	This work
**Plasmids**		
pVWEx1	Kan^R^, *C. glutamicum/E. coli* shuttle vector (P_tac_, *lacI*, pHM1519 oriV_C.g._)	^85^
pEKEx3	Spec^R^, *C. glutamicum/E. coli* shuttle vector (P_tac_, *lacI*, pBL1 OriV_C.g._)	^86^
pECXT99A	Tet^R^, *C. glutamicum/E. coli* shuttle vector (P_trc_, *lacI*, pGA1 OriV_C.g._)	^87^
pVWEx1-*dpkA*	Kan^R^, pVWEx1 overexpressing *dpkA* from *P. putida* KT2440 and change of start codon GTG to ATG	This work
pEKEx3-*xylA*_*Xc*_*-XylB*_*Cg*_	Spec^R^, pEKEX3 overexpressing *xylA* from *Xanthomonas campestris* SCC1758 and *xylB* from *C. glutamicum* ATCC 13032	^45^
pECXT99A-*araBAD*	Tet^R^, pECXT99A overexpressing *araBAD* from *E. coli* MG1655	This work
pECXT99A-*amyA*	Tet^R^, pECXT99A overexpressing *amyA* from *Streptomyces griseus* IMRU3570	^42^

### Fed-Batch cultivation

Fermentation of *C. glutamicum* NMeAla1 was performed in an initial working volume of 4 L in a bioreactor (7 L NLF, Bioengineering AG, Switzerland) at 30 °C, 0.2 bar overpressure, and an aeration rate of 5 NL min^−1^. Stirrer speed was controlled to maintain relative dissolved oxygen saturation at 30% during growth phase. Due to controlled addition of KOH (4 m) and phosphoric acid (10% (w/w)) the pH was maintained at 7.0. To avoid foaming the antifoam Struktol® J647 was added manually when necessary. The first feeding phase with 26.7 g L^−1^ potassium acetate solution (total volume: 500 mL) was depending on the relative dissolved oxygen saturation, it was activated when the rDOS signal rose above 60% and stopped when rDOS felt below 60%. The second feeding phase (164 g L^−1^ glucose and 84 g L^−1^ MMA (total volume: 1000 mL)) was started manually after 22 h. Samples were taken automatically every 2 hours within the first 24 h and every 8 hours afterwards and cooled to 4 °C until analysis. For fermentation a modified CGXII minimal medium was used: 5 g L^−1^ (NH_4_)_2_SO_4_, 1.25 g L^−1^ urea, 1 g L^−1^ K_2_HPO_4_, 1 g L^−1^ KH_2_PO_4_, 5 g L^−1^ yeast extract in addition to the same concentrations of trace elements and vitamins as described elsewhere^18^. Modified CGXII was supplemented with 15 g L^−1^ KAc, 20 g L^−1^ glucose, 9.3 g L^−1^ MMA and 25 µg mL^−1^ kanamycin. The fermenter was inoculated by addition of 450 mL of a shake flask culture grown in the described media with extra 42 g L^−1^ MOPS buffer.

### Molecular genetic techniques and strain construction

The standard molecular genetic techniques were performed as described in Grenn and Sambrook, 2012. Transformation of *E. coli* DH5α^78^ was performed by heat shock^79^, plasmid DNA transfer into *C. glutamicum* by electroporation^22^.The gene *dpkA* was amplified from *P. putida* KT2440 genomic DNA by using the primers dpkA-fw (GCCAAGCTTGCATGCCTGCA*GAAAGGAGGCCCTTCAG*ATGTCCGCACCTTCCACCAG) and dpkA-rv (GGGATCCTCTAGAGTCGACCTGCATCAGCCAAGCAGCTCTTTCA); dpkA-fw carries the RBS sequence (italicized). For higher expression rates the start codon of *dpkA* was changed from GTG to ATG (underlined). The vector pVWEx1 was restricted with BamHI and incubated in a Gibson assembly^80^ with the PCR product for construction of plasmid pVWEx1-*dpkA* which was used to transform *C. glutamicum* strains. For construction of the expression plasmid harboring the genes for arabinose degradation *araBAD* from *E. coli* was amplified using genomic DNA of *E. coli* MG1655 with the primers araBAD-fw (CATGGAATTCGAGCTCGGTACCCGGG*GAAAGGAGGCCCTTCAG*ATGGCGATTGCAATTGGCCT) and araBAD-rv (GCCTGCAGGTCGACTCTAGAGGATCTTACTGCCCGTAATATGCCT); araBAD-fw carries the RBS sequence (italicized). The vector pECXT99A was incubated with BamHI for restriction and incubated with the PCR product in an Gibson assembly^80^ for plasmid construction. The constructed plasmid was used to transform *C. glutamicum* strains.

### Crude extract preparation and enzyme assays

Cells for crude extracts were inoculated as described above and harvested after 20 h and stored at −20 °C. From this step cell pellets and crude extract were handled at 4 °C or on ice. 150 to 200 mg cells were resuspended in 1 mL 100 mm glycine buffer (pH 10) and sonicated (UP 200 S, Dr. Hielscher GmbH, Teltow, Germany) at an amplitude of 60% and a duty cycle of 0.5 for 9 min. Protein concentration of the cell free extracts obtained by centrifugation (20200 × g, 30 min, 4 °C) was determined by the Bradford method^81^ with bovine serum albumin as reference.

For determination of the reductive *N*-methylamination activity the assay was performed as described^18^. In a total volume of 1 mL containing 100 mm glycine buffer (pH 10), 60 mm MMA, 10 mm pyruvate and 0.3 mm NADPH the consumption of NADPH (epsilon = 6200 L mol^−1^ cm^−1^) was detected at 340 nm at 30 °C for 3 min. The assay was performed in at least triplicates.

### Quantification of amino acids and organic acids

Extracellular amino acids and pyruvate were quantified by high-performance liquid chromatography (HPLC) (1200 series, Agilent Technologies Deutschland GmbH, Böblingen, Germany). The culture supernatants were collected and centrifuged (20200 × g, 15 min) for further analysis.

For the detection of NMeAla and l-alanine the samples were derivatised with 9-fluorenylmethyl chlorocarbonate (Fmoc-Cl) according to published methods^82^ with modifications^39^. l-proline was used as internal standard. The separation was carried out by a reversed phase HPLC using a pre-column (LiChrospher 100 RP8 EC-5 μ (40 mm × 4.6 mm), CS-Chromatographie Service GmbH, Langerwehe, Germany) and a main column (LiChrospher 100 RP8 EC-5 μ (125 mm × 4.6 mm), CS Chromatographie Service GmbH). The detection was performed with a fluorescence detector (FLD G1321A, 1200 series, Agilent Technologies) with the excitation and emission wavelength of 263 nm and 310 nm respectively.

Analysis of l-valine was performed by an automatic pre-column derivatization with ortho-phthaldialdehyde (OPA)^83^ and separated on a reversed phase HPLC using pre- and main column (LiChrospher 100 RP8 EC-5μ, 125 mm × 4.6 mm, CS Chromatographie Service GmbH) with l-asparagine as internal standard. Detection of the fluorescent derivatives was carried out with a fluorescence detector with an excitation wavelength of 230 nm and an emission wavelength of 450 nm. Concentrations exceeding 0.1 g L^−1^ were considered further.

Pyruvate, acetate and glucose concentrations were measured with an amino exchange column (Aminex, 300 mm × 8 mm, 10 μm particle size, 25 Å pore diameter, CS Chromatographie Service GmbH) under isocratic conditions for 17 min at 60 °C with 5 mm sulfuric acid and a flow rate of 0,8 mL min^−1^. The detection was carried out with a Diode Array Detector (DAD, 1200 series, Agilent Technologies) at 210 nm. Concentrations exceeding 0.5 g L^−1^ were considered further.

### Transcriptome analysis using DNA microarrays

For the transcriptome analysis in the presence of MMA, *C. glutamicum* wild type cells were grown in minimal medium supplemented with 250 mm MMA or 125 mm ammonium sulfate to exponential growth phase and harvested at an OD_600_ of 4. The RNA was isolated and transcriptome analysis using whole genome microarrays were performed as described previously^84^.

## Electronic supplementary material

Supplementary Dataset 1

## Data Availability

All data generated or analyzed during this study are included in this published article (and its Supplementary Information files).
