# Supplementary material for: One-step process for production of N-methylated amino acids from sugars and methylamine using recombinant Corynebacterium glutamicum as biocatalyst
Source: Sci Rep. 2018 Aug 27;8:12895. doi: 10.1038/s41598-018-31309-5 (PMC6110843; doi:10.1038/s41598-018-31309-5)
Supplement: Supplementary file 1 — Supplementary Dataset 1 [file 41598_2018_31309_MOESM1_ESM.pdf]

Supplementary Data Set

One-step process for production of *N*-methylated amino acids from sugars and methylamine using recombinant *Corynebacterium glutamicum* as biocatalyst

Melanie Mindt[a], Joe Max Risse[b], Hendrik Gruß[c], Norbert Sewald[c], Bernhard J. Eikmanns[d] and Volker F. Wendisch[a,#]

[a] Genetics of Prokaryotes, Faculty of Biology & CeBiTec, Bielefeld University, Universitätsstr. 25, 33615 Bielefeld, Germany

[b] Fermentation Technology, Technical Faculty & CeBiTec, Bielefeld University, Universitätsstr. 25, 33615 Bielefeld, Germany

[c] Organic Chemistry III, Faculty of Chemistry & CeBiTec, Bielefeld University, Universitätsstr. 25, 33615 Bielefeld, Germany

[d] Institute of Microbiology and Biotechnology, University of Ulm, Albert-Einstein-Allee 11, 89081 Ulm, Germany

[#]Corresponding author: Volker F. Wendisch, Chair of Genetics of Prokaryotes, Faculty of Biology & CeBiTec, Bielefeld University, Germany; phone: +49-521-106 5611; fax: +49-521-106 5626; volker.wendisch@uni-bielefeld.de

Supplementary Table: Differential gene expression of *C. glutamicum* wild type grown in glucose containing CGXII minimal medium supplemented with 250 mM MMA or 125 mM $(NH_4)_2SO_4$.

Gene ID[ab]	Gene name[b]	Gene annotation[b]	mRNA level $(MMA/(NH_4)_2SO_4)$[c]
cg0759	*prpD2*	2-Methylcitrate dehydratase, involved in propionate catabolism	5.0
cg0762	*prpC2*	2-Methylcitrate synthase, involved in propionate catabolism	2.7
cg0801	-	Hypothetical protein	2.7
cg2566	-	Putative secreted protein	0.3
cg3402	-	Putative Hg_2^+ permease, MerTP-family	2.7

[a] Genes shown are sorted to their identifiers.

[b] Gene ID, name and annotation are according to BX927147

[c] Differential gene expression as calculated for two biological replicates. Values listed were selected for $P < 0.05$ and at least twofold change of the RNA level.
